# Spontaneous Coronary Artery Dissection: A Case Series Reviewing Typical Presentations of an Atypical Pathology

**DOI:** 10.5811/cpcem.59930

**Published:** 2023-11-08

**Authors:** Jace C. Bradshaw, Lisa Saffire, Jake Valentine, P. Logan Weygandt

**Affiliations:** * Johns Hopkins Hospital, Department of Anesthesiology and Critical Care Medicine, Baltimore, Maryland; † Johns Hopkins Hospital, Department of Emergency Medicine, Baltimore, Maryland; ‡ HCA Houston Healthcare Kingwood, Department of Emergency Medicine, Kingwood, Texas

**Keywords:** case report, myocardial infarction, spontaneous coronary artery dissection, ECG

## Abstract

**Introduction:**

Spontaneous coronary artery dissection (SCAD) is a rare cause of myocardial infarction. Patients suffering SCAD are often young women without typical risk factors for atherosclerotic heart disease. Clinicians should maintain a high index of suspicion for SCAD.

**Case series:**

We report three cases of patients with SCAD from a single physician in a six-month period. Each case is unique and highlights the varied presentations and epidemiological risk factors associated with this condition.

**Discussion:**

We believe these cases are unique in that they provide insights into the variable presentations and conditions frequently associated with SCAD and will help clinicians maintain a high index of suspicion for this difficult to diagnose and rare cause of type 2 myocardial infarction. We discuss differences in interventional techniques and medical management.

CPC-EM Capsule What do we already know about this clinical entity?

*Spontaneous coronary artery dissection (SCAD) occurs when an epicardial coronary artery dissects without a clear precipitant.*

What makes this presentation of disease reportable?

*Each case in this series highlights the varied presentations and epidemiological risk factors associated with SCAD.*

What is the major learning point?

*Spontaneous coronary artery dissection is a rare cause of myocardial infarction, most common in young women without typical risk factors for heart disease.*

How might this improve emergency medicine practice?

*Clinicians should maintain a high index of suspicion for this pathology. We discuss differences in interventional techniques and medical management.*

## INTRODUCTION

Spontaneous coronary artery dissection (SCAD) is an uncommon cause of myocardial infarction (MI) without underlying acute coronary atherothrombosis, often occurring in individuals with few atherosclerotic risk factors.^1^ Spontaneous coronary artery dissection occurs when an epicardial coronary artery dissects without a clear precipitant as in penetrating atherosclerotic plaque or trauma. Vessel dissection may result in coronary artery obstruction caused by formation of an intramural hematoma or intimal disruption at the site of dissection—resulting in myocardial ischemic injury.^1^

Knowledge of the epidemiology and risk factors can heighten a clinician’s suspicion for SCAD in the appropriate patient population, leading to early diagnosis and possibly improved prognosis. In this case series, we discuss three patients who presented with SCAD to a single physician over a six-month period, each illustrating key points related to the natural history of the disease. This case series is unique as it presents a spectrum of risk factors and patient presentations that emergency physicians may encounter; we also discuss management strategies. All patients provided written informed consent.

## CASE SERIES

### Case 1

A 43-year-old female with a history of migraines presented to an urban, university-affiliated emergency department (ED) after she developed unrelenting chest pain and shortness of breath. The pain began following her normal exercise routine, and she reported that the pain radiated to her back and down her left arm. She was hypertensive on arrival in the ED, including blood pressure of 149/90 millimeters of mercury (mm Hg) and a heart rate of 92 beats per minute. She was noted to be uncomfortable and in moderate distress.

**Image 1.**
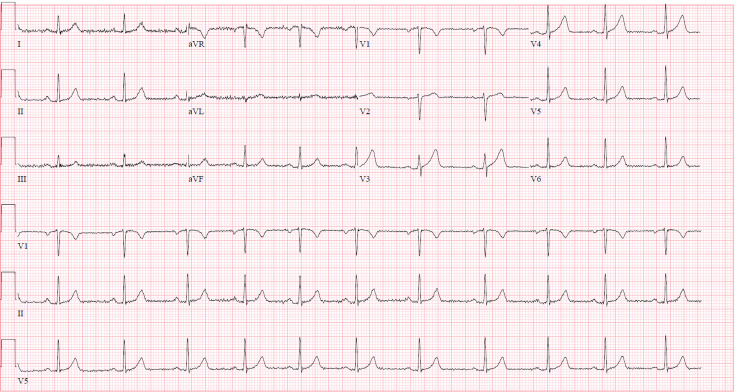
Sinus rhythm with prominent T-waves in leads V3–V4.

Based on her chief complaint, an electrocardiogram (ECG) was performed on arrival that demonstrated prominent precordial t-waves. The differential diagnosis for those findings included hyperacute T-waves, benign early repolarization, and pericarditis (Image 1). Serial ECGs remained unchanged. Laboratory work-up revealed elevated cardiac enzymes: her initial troponin-I resulted at 0.12 nanograms per milliliter (ng/ml) (normal troponin-I < 0.03 ng/ml). Despite having no conventional risk factors, the patient’s unremitting chest pain and elevated cardiac biomarkers were concerning for ongoing acute coronary syndrome (ACS).While her ECGs did not evolve during her diagnostic work-up, her troponin-I continued to rise, peaking at 20.8 ng/ml. She was taken for urgent angiography, which demonstrated SCAD of the left anterior descending artery (LAD), originating just after the origin of the first diagonal artery and continuing to the apex.

Based on the catheterization findings, the decision was made to continue medical management: 72 hours of anticoagulation followed by a year of dual antiplatelet therapy (DAPT). Echocardiography showed normal left ventricular function with a normal ejection fraction (EF); no apical wall motion abnormalities were identified. Her chest pain resolved, and she was discharged in stable condition on DAPT and beta-blockers.

### Case 2

A 45-year-old female with a history of hypertension, fibromuscular dysplasia, and cerebral aneurysm with previous clipping presented to the ED after severe non-radiating chest pain awakened her from sleep. On arrival, the patient was hypertensive (150/91 mm Hg), but the remainder of her vital signs were normal. While in the ED, she began experiencing dyspnea and nausea. She was noted to be uncomfortable, diaphoretic, and pale, but no cardiopulmonary abnormalities were identified on physical exam.

**Image 2.**
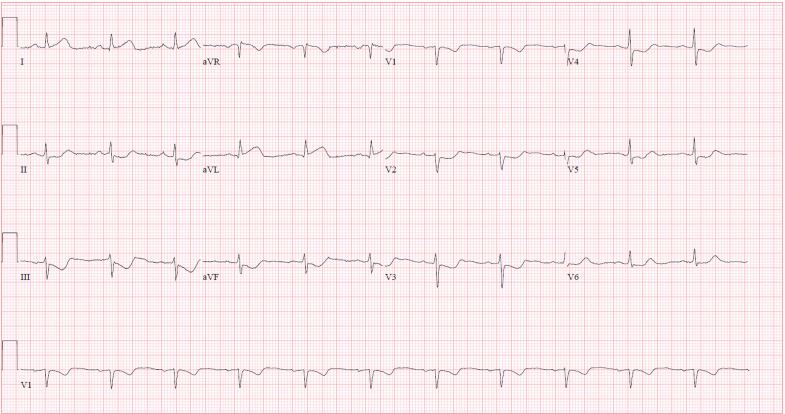
Sinus rhythm with ST-segment elevation in leads I and aVL and ST segment depression in leads II, III, aVF, and V2-V5. This electrocardiogram is concerning for high-lateral infarction involving the left anterior descending or left circumflex artery.

Her ECG on arrival demonstrated ST-segment depression in leads V2–V6 and subtle ST-segment depression/T-wave inversions in the inferior leads. She continued to have unrelenting chest pain, and her repeat ECG showed ST-segment elevation in leads I and aVL concerning for a high-lateral ST-segment elevation myocardial infarction (STEMI) (Image 2). Nitroglycerin administration provided little improvement of her symptoms; she received aspirin, ticagrelor, and anticoagulation with heparin infusion.

She was taken for emergent left heart catheterization, which demonstrated SCAD of a small diagonal branch of the mid-LAD. There were no lesions amenable to percutaneous coronary intervention (PCI). After catheterization, her chest pain resolved, and her troponin-I peaked at 23 ng/ml. Echocardiography showed normal ventricular function without significant wall motion abnormality. She was discharged on an aggressive regimen of blood pressure control that included rate-control agents to prevent propagation of the dissected area.

### Case 3

**Image 3.**
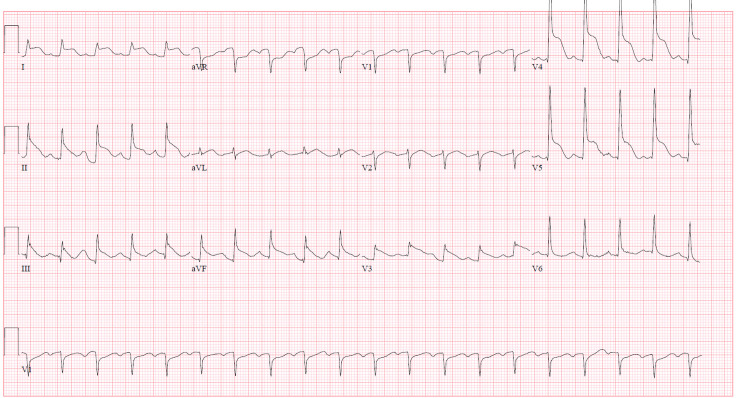
Sinus rhythm with ST-segment elevations in leads I, II, III, aVF, and V3-V5 as well as ST-segment depression in aVR and V1. This electrocardiogram is concerning for a large territory. left anterior descending artery infarction with probable left circumflex artery involvement.

A 33-year-old female, who was two weeks postpartum after cesarean section for preterm premature rupture of membranes, was brought to the ED by emergency medical services (EMS) for crushing chest pain. The initial ECG provided by EMS showed a sinus tachycardia with no signs of ischemia or ST-segment changes. On arrival to the ED, the patient had an episode of seizure-like activity and became unresponsive.Advanced Cardiac Life Support was initiated after the patient was found to be pulseless with agonal respirations. During the first rhythm check, the patient’s ECG demonstrated ventricular fibrillation. After two attempts at defibrillation, return of spontaneous circulation was obtained. Her post-arrest ECG showed anterolateral and inferior ST segment elevation (Image 3).

During this ongoing resuscitation, the case was discussed with the multidisciplinary Heart Attack Team at our institution, and the cardiothoracic surgery service was alerted for possible extracorporeal membrane oxygenation cannulation. The interventional cardiology service agreed to take the patient for emergent PCI but maintained a high suspicion for SCAD.The patient was found to have SCAD from the ostium of the LAD to the apex, involving nearly all the diagonal branches. The LAD appeared to be 80% stenosed from intrusion of the ostium of the LAD into the true lumen of the vessel. Percutaneous coronary intervention was avoided in the setting of demonstrated thrombolysis in myocardial infarction (TIMI)-3 flow and the increased risk that revascularization posed of propagating the dissection. The patient’s care team elected for medical management in the cardiac intensive care unit (CCU).

In the CCU, her ST-segment elevation resolved, and the patient became free of chest pain. Echocardiography demonstrated reduced left ventricular function (EF 25–30%) with apical ballooning. Four days later the patient developed recurrent chest pain, anterior ST-segment elevation and an elevated troponin-I, which peaked at 60.92 ng/ml. Repeat left heart catheterization did not show any changes; so aggressive medical optimization continued. Nine days after presentation, the patient was discharged from the hospital free of chest pain and neurologically intact. She was discharged on goal-directed medical therapy for heart failure with reduced EF. A repeat echocardiogram 40 days after discharge demonstrated improvement in ventricular wall motion, and one year later her EF was stable at 50–55%.

## DISCUSSION

Spontaneous coronary artery dissection is a rare condition; however, because diagnosis requires coronary angiography, it is likely under-reported. This condition occurs predominantly in young women who often have no risk factors for atherosclerotic heart disease, and they can present with varied symptoms and ECG patterns that span from normal to ST-segment elevation to ventricular dysrhythmias.^1^ Myocardial infarction related to SCAD is considered type 2 as it leads to an imbalance in myocardial oxygen supply and demand without the presence of atherosclerotic plaque rupture.^2^

Prevalence has been reported between 0.3–4% among patients undergoing routine and urgent angiography, respectively.^3^ Spontaneous coronary artery dissection is the underlying cause of chest pain in up to 10% of women less than 50 years of age, and SCAD is seen in up to 35% of cases with elevated cardiac biomarkers in this cohort.^1^ ^,^ ^4^ Furthermore, SCAD is the most common cause of MI in pregnant women, making up 43% of these cases.^5^ As demonstrated by these findings, it is important to have a high index of suspicion for SCAD in patients presenting with symptoms suggestive of ACS who lack the typical risk factors. Each of the above cases demonstrates a risk factor and potential presentation for this rare condition. Demographic factors of SCAD can be found in Table 1.

**Table 1.** Demographic factors of patients with spontaneous coronary artery dissection (SCAD). This data is based on data queried from the Mayo Clinic “Virtual” Multicenter SCAD Registry composed of 1,196 patients.^6^

Age, median (IQR)	54 (47–61)
Gender, %	
Female	95.6
Male	3.9
BMI, median (IQR)	25.0 (21.8–29.2)
Race, %	
White	92.3
Black	2.3
Hispanic/Hispanic-White	2.2
Other	3.2
Comorbidities, %	
Classic ACS risk factors	
Hypertension	32.2
Hyperlipidemia	33.0
Diabetes mellitus	2.9
Previous tobacco use, *n* (%)	26.4

*IQR*, interquartile range; *BMI*, body mass index; *ACS*, acute coronary syndrome.

The first patient presented with non-specific ECG changes and an elevated troponin-I. Normal or non-specific ECG findings occur in 46% of observed SCAD patients, and troponin elevations occur in approximately 80% of cases.^6^ Additionally, this patient suffered from migraine headaches, which is more prevalent in patients with SCAD than those without.^7^ Migraine headaches are more commonly associated with SCAD in the setting of pregnancy.^8^ It remains unclear whether migraine is an independent risk factor for SCAD, but the epidemiology of patients who suffer from both migraine and SCAD is unique. Further study is needed to definitively elucidate the relationship between migraine and vascular conditions such as SCAD.^7^ Despite this association, this patient was still assumed to have atherothrombosis and managed accordingly. Her diagnosis, made on urgent angiography after admission, highlights the diagnostic uncertainty that emergency physicians encounter when managing a patient with ACS.

The second patient presented with an ECG that met STEMI criteria. In patients with SCAD, 39–49% had ECGs diagnostic of STEMI.^3^ ^,^ ^6^ The precordial leads (V1–V4) most frequently demonstrate ST-segment elevation, corresponding to the LAD territory.^3^ However, SCAD can involve multiple vessels and, as a result, multiple ECG territories.^1^ This patient had a history of fibromuscular dysplasia (FMD). Fibromuscular dysplasia is a vascular disease that affects medium-sized arteries: most commonly affecting the renal, carotid, and vertebral arteries, but it has been known to affect the coronary arteries.^9^ A build-up of fibrous tissue and webs in the arterial walls causes arterial stenosis, tortuous arteries, aneurysms, and dissections.^9^

Fibromuscular dysplasia is the most common nonatherosclerotic phenomenon found to cause SCAD.^1^ A 2013 prospective and retrospective study in Vancouver screened 86% of patients identified with nonatherosclerotic SCAD for FMD, and found 72% of these patients to have FMD.^10^ Other studies have found the association to be weaker: a study using the Mayo Clinic’s registry of patients with confirmed SCAD showed that approximately 39% were confirmed positive for FMD; however, 31% of patients included in the study were not screened at all.^6^

The final patient presented with chest pain, followed by cardiac arrest, and was ultimately found to have an ECG that met STEMI criteria. Chest pain is the presenting symptom in approximately 80–90% of SCAD cases.^1^ ^,^ ^6^ ^,^ ^11^ Ventricular arrythmias, which this patient presented with, were noted in 3–16% of cases.^1^ ^,^ ^6^ ^,^ ^11^ Of the cases with ventricular arrythmias, 48% were diagnosed with STEMI after defibrillation.^12^ While acute atherothrombosis is uncommon in pregnant and postpartum women, SCAD shows a well-defined correlation with late pregnancy and the postpartum period.^3^ Clinicians should be aware of this potential diagnosis in pregnant and postpartum patients who present with chest pain.

Overall, initial patient management is similar to management of atherosclerotic ACS: focus should be on revascularization of ischemic myocardial territories, at least until the definitive diagnosis of SCAD is made.^13^ ^,^ ^14^ Compared with the revascularization approach in atherosclerotic ACS, restoration of TIMI 3 flow with as few interventional measures as possible is the goal in management of SCAD—even if coronary architecture is not normalized.^14^

Medical management of patients suffering from SCAD differs from management of atherosclerotic ACS or MI. While there is limited evidence, current guidelines indicate that systemic anticoagulation is unnecessary and may propagate the intramuscular hematoma; therefore, it should not be implemented or discontinued in the absence of other indications for systemic anticoagulation if SCAD is suspected.^1^ ^,^ ^14^ Similarly, there is limited evidence to guide the use of anti-platelet agents such as glycoprotein IIb/IIIa inhibitors in the emergency management of SCAD.^1^ ^,^ ^14^ Theoretical concerns about the extension of dissection and additional bleeding risk limit the use of antiplatelet agents if SCAD is suspected in the ED.^1^ ^,^ ^14^

Beta-blocker therapy to reduce shear stress is supported by one retrospective study^14^ that showed an association with lower rates of SCAD recurrence, but these results have not been replicated.^15^ Decisions regarding initiation of systemic rate-control agents to reduce shear stress, anticoagulation, and/or DAPT in the emergency setting should be made in conjunction with an interventional cardiologist when available if concern for SCAD is high. While nuanced management of SCAD is better reserved for subspecialty discussion, it is important for the emergency physician to understand the potential harms of antiplatelet agents, anticoagulation, and revascularization interventions in this population.

While there are other cases of SCAD in the literature highlighting risk factors for this increasingly recognized entity,^16^ we believe the cases presented here illustrate the varied presentations and important risk factors associated with this uncommon and difficult-to-diagnose condition. Clinicians should be aware of the disease’s natural history, as early diagnosis and intervention may lead to improved outcomes.^15^ ^,^ ^16^

## CONCLUSION

Spontaneous coronary artery dissection is a rare cause of type 2 myocardial infarction that affects primarily young women who differ in risk profile when compared with patients who suffer type 1 MI. Associated factors include migraine, connective tissue disorders, and pregnancy. Presentations are varied, and clinicians must maintain a high suspicion for this uncommon condition.
